# Supplementary material for: Selective Degradation Permits a Feedback Loop Controlling Annexin A6 and Cholesterol Levels in Endolysosomes of NPC1 Mutant Cells
Source: Cells. 2020 May 7;9(5):1152. doi: 10.3390/cells9051152 (PMC7291204; doi:10.3390/cells9051152)
Supplement: Supplementary file 1 [file cells-09-01152-s001.pdf]

cells

Supplementary materials

Selective degradation permits a feedback loop controlling Annexin A6 and cholesterol levels in endolysosomes of NPC1 mutant cells

Elsa Meneses-Salas, Ana García-Melero, Patricia Blanco-Muñoz, Jaimy Jose, Marie-Sophie Brenner, Albert Lu, Francesc Tebar, Thomas Grewal, Carles Rentero and Carlos Enrich

Supplementary Materials

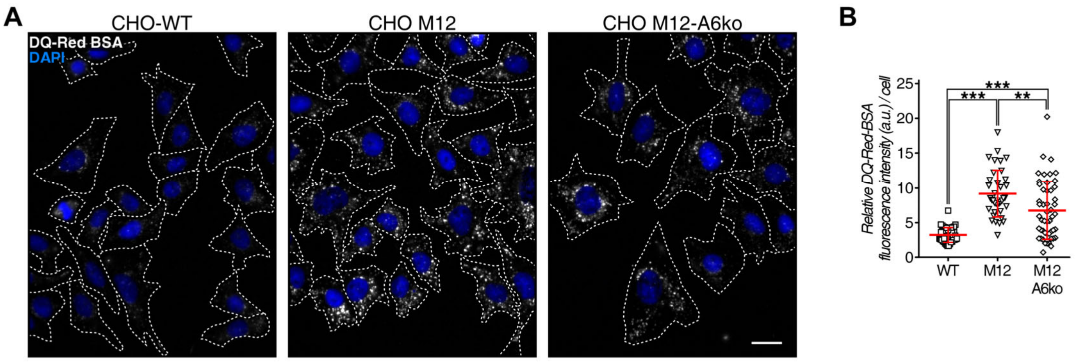

Figure S1. AnxA6 depletion normalizes increased capacity for bulk lysosomal degradation in NPC1 mutant cells. (A) CHO-WT, CHO M12, and CHO M12-A6ko cells were incubated with 10 µg/ml DQ-Red-BSA (gray) for 6 h at 37°C. Cells were fixed, nuclei were stained with DAPI (blue), and cells were visualized in a confocal microscope. Merged images are representative for three independent experiments. For better comparison of DQ-Red-BSA staining, the outline and shape of cells is indicated. Scale bar is 20 µm. (B) Quantification of the bright red (pseudocolored grey) fluorescent signal (Total cell fluorescence; arbitrary units, a.u.) derived from internalized and cleaved DQ-Red-BSA in late endosomes/lysosomes served as a measure for bulk lysosomal degradation (see Material and Methods for further details) [41]. More than 50 cells per cell line in three separate experiments were analyzed. The data is shown as dot plot. The mean ± SEM is also given (**$p < 0.01$, ***$p < 0.001$).

© 2020 by the authors. Submitted for possible open access publication under the terms and conditions of the Creative Commons Attribution (CC BY) license (http://creativecommons.org/licenses/by/4.0/).
